# Multimodal treatment approach with sunitinib, pembrolizumab, bevacizumab, sirolimus, and zoledronic acid for locally advanced clear cell renal cell carcinoma: a case report

**DOI:** 10.3325/cmj.2025.66.299

**Published:** 2025-08

**Authors:** Mahdi Mehrabi, Mohammadreza Eslami, Mehrdad Payandeh

**Affiliations:** 1 Student Research Committee, Kermanshah University of Medical Sciences, Kermanshah, Iran; 2 Internal Medicine Department, School of Medicine, Kermanshah University of Medical Sciences, Kermanshah, Iran

## Abstract

Locally advanced renal cell carcinoma (RCC) presents significant therapeutic challenges, particularly in resource-limited settings with restricted access to new therapies. This report describes a new exploratory multimodal therapeutic approach for a patient with locally advanced clear cell RCC (ccRCC) with adrenal and lymph node metastases. A 45-year-old woman presented with an incidentally discovered 9-cm mass in the left kidney, which was later diagnosed as grade-2 ccRCC with adrenal and lymph node involvement. After radical nephrectomy, a multimodal treatment regimen consisting of sunitinib, pembrolizumab, bevacizumab, sirolimus, and zoledronic acid was initiated. Following eight cycles of treatment, computed tomography imaging and ultrasound showed considerable tumor shrinkage, with residual mass decreasing from 22 × 42 mm to 22 × 18 mm, which constituted a “radiologically complete response.” The patient had compensatory hypertrophy of the contralateral kidney with preserved renal function. This case illustrates the potential efficacy of a novel multimodal treatment strategy combining targeted therapies, immunotherapy, and bone-modifying agents in a resource-limited setting. Further research is needed to validate this approach in larger, diverse patient cohorts.

Renal cell carcinoma (RCC) is the 14th most common malignancy worldwide, with clear cell RCC (ccRCC) accounting for 70% of cases (1). The treatment of locally advanced ccRCC, particularly involving adrenal glands and lymph nodes, is complex and requires innovative approaches. Recent clinical trials have demonstrated the efficacy of combining immune checkpoint inhibitors with vascular endothelial growth factor (VEGF) inhibitors in advanced RCC (2). However, these therapies remain inaccessible in many resource-limited settings due to economic and logistical constraints.

This case report describes an exploratory multimodal treatment regimen that combines five agents: sunitinib (VEGF tyrosine kinase inhibitor), pembrolizumab (programmed death-ligand 1 [PD1] inhibitor), bevacizumab (anti-VEGF monoclonal antibody), sirolimus (mammalian target of rapamycin [mTOR] inhibitor), and zoledronic acid (bone-modifier). This approach represents a potential new strategy to enhance therapeutic benefit in patients with locally advanced ccRCC in health care settings with limited access to newer anticancer drugs.

## Case report

### Patient information

This case study is structured according to the CAse REport guidelines (3). A 45-year-old woman was referred for evaluation at Imam Reza Hospital, Kermanshah, in August 2023 after a routine health examination incidentally discovered a mass in the left kidney. The patient had no significant medical history and reported no cardinal symptoms of RCC, including hematuria, flank pain, or weight loss.

### Clinical and diagnostic findings

**Figure 1:**
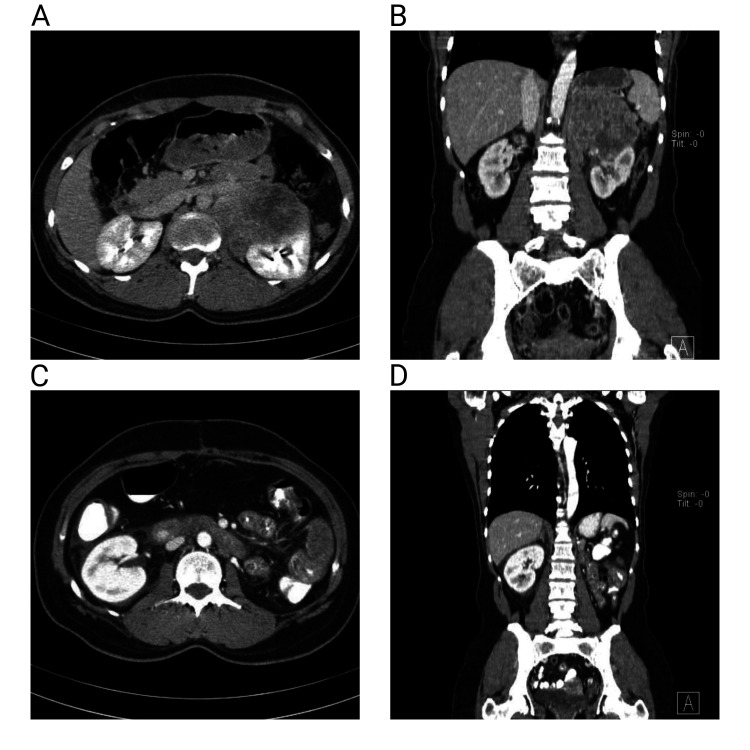
Treatment response assessment by computed tomography (CT) in a patient with left-sided clear cell renal cell carcinoma (ccRC). Pre-treatment scans: (**A**) axial view showing a large, heterogeneous, well-defined mass originating from the left kidney; (**B**) coronal view confirming the extent of the left renal mass. Post-treatment scans: (**C**) axial view and (**D**) coronal view obtained at similar anatomical levels demonstrate marked interval reduction in the size of the mass, consistent with an effective therapeutic intervention.

Physical examination revealed a firm, non-tender mass in the left upper quadrant, with no evidence of palpable lymphadenopathy or lower extremity edema. Vital signs were within reference ranges. Abdominal and pelvic computed tomography (CT) identified a 9-cm heterogeneous mass in the left kidney extending into the adrenal gland and perinephric fat, accompanied by enlarged para-aortic lymph nodes (Figure 1). A chest CT scan showed no pulmonary metastases, though abdominal imaging confirmed bone metastases.

### Diagnostic assessment

Post-nephrectomy pathological examination confirmed grade-2 ccRCC with invasion into the adrenal gland, perinephric fat, hilar lymphatics, and lymphatic/perineural invasion. Standard diagnostic pathway with CT imaging and pathological confirmation was followed without significant delays. The final diagnosis was locally advanced grade-2 ccRCC with adrenal, lymph node, and bone metastases. Differential diagnosis considered renal masses, but imaging and pathology confirmed ccRCC. Unfavorable factors at diagnosis included grade-2 histology, locally advanced stage (adrenal/lymph node involvement), bone metastases, and lymphatic/perineural invasion, indicating a higher risk of recurrence and poorer prognosis.

### Therapeutic intervention

The patient underwent left radical nephrectomy with adrenalectomy and lymph node dissection. Pathological analysis confirmed a 9-cm grade-2 ccRCC with invasion into the adrenal gland, perinephric fat, and hilar lymphatics. Histological features included an acinar pattern with lymphatic and perineural invasion.

Subsequently, a multimodal treatment regimen was initiated, comprising: 1) sunitinib: 25 mg orally, two weeks on, one week off, targeting VEGF-mediated angiogenesis; 2) pembrolizumab: 200 mg intravenously every three weeks, boosting immune recognition via PD-1 inhibition; 3) bevacizumab: 400 mg intravenously every three weeks, specifically blocking VEGF-A to disrupt tumor vascularization; 4) sirolimus: 1 mg orally daily, inhibiting the mTOR pathway critical to ccRCC cell proliferation; and 5) zoledronic acid: 4 mg intravenously every three weeks, addressing bone metastases and preventing skeletal complications. This regimen spanned eight three-week cycles, with close monitoring for efficacy and toxicity.

### Follow-up and outcomes

After the major nephrectomy surgery, the patient declined further invasive procedures such as biopsy, and our primary method of follow-up was imaging. Post-treatment imaging showed significant tumor regression, with the residual mass shrinking from 22 × 42 mm to 22 × 18 mm. Compensatory hypertrophy of the contralateral kidney was noted (length 120 mm), and laboratory tests indicated normal renal function. No bladder lesions were observed. While the overall toxicity profile was considered manageable and allowed the patient to complete the planned treatment duration, the patient did experience several anticipated adverse events. These were graded according to the Common Terminology Criteria for Adverse Events, version 5.0, as follows: oral mucositis, corresponding to a maximum of grade 2 (moderate pain limiting instrumental activities of daily living (ADL) related to oral intake moderate oral ulcers or erythema present); anorexia, corresponding to a loss of appetite, consistent with grade 1 (loss of appetite without alteration in eating habits); fatigue not fully relieved by rest, which moderately interfered with the patient’s usual activities, consistent with grade 2 (moderate fatigue; limiting instrumental ADL); and anemia (a decrease in hemoglobin, with the lowest level recorded at 10.0 g/dL, corresponding to grade 1). All the observed adverse events were managed effectively with supportive care measures. The timeline of events and interventions is shown in Table 1.

**Table 1:** The timeline of events and interventions

Event description	Date
• CT scan performed to investigate a left renal mass incidentally discovered during a routine health exam. • CT revealed a 9-cm left kidney mass with adrenal and perinephric fat extension, enlarged para-aortic lymph nodes, and bone metastases, indicating locally advanced ccRCC.	**August 6, 2023** **Diagnostic CT scan for incidental renal mass**
• Surgical removal of the left kidney, adrenal gland, and regional lymph nodes, aiming for maximum cytoreduction.	**August 14, 2023** **Left radical nephrectomy, adrenalectomy, and lymph node dissection**
• Pathology confirmed a grade-2 ccRCC with invasion into the adrenal gland, perinephric fat, and hilar lymphatics, including lymphatic and perineural invasion.	**August 18, 2023** **Pathology findings**
• Start of a novel multimodal regimen: sunitinib, pembrolizumab, bevacizumab, sirolimus, and zoledronic acid (eight 3-week cycles). • The treatment was designed as an exploratory approach to target angiogenesis, immune evasion, mTOR pathway, and bone metastases in a resource-limited setting post-nephrectomy for a locally advanced disease.	**September 4, 2023** **Multimodal adjuvant therapy initiated**
• A follow-up CT scan performed after several treatment cycles demonstrated tumor shrinkage, with residual mass decreasing from 22×42mm to 22×18mm, which indicated treatment response.	**November 19, 2023** **Interim CT scan results**
• After eight multimodal treatment cycles, a CT scan confirmed tumor size reduction with no evidence of disease progression.	**February 28, 2024** **Final post-treatment CT scan results**
• Ultrasound examination confirmed the absence of a detectable disease, indicating a complete radiological response based on imaging and clinical assessment. Patient had compensatory contralateral kidney hypertrophy and preserved renal function throughout treatment.	**April 15, 2024** **Ultrasound findings consistent with “radiological response”**

*Abbreviations: CT – computed tomography, ccRCC –clear cell renal cell carcinoma; mTOR – mammalian target of rapamycin.

## Discussion

Locally advanced ccRCC poses significant treatment challenges, especially in resource-limited settings where advanced therapies are scarce. This report presents an exploratory regimen combining sunitinib, pembrolizumab, bevacizumab, sirolimus, and zoledronic acid that achieved tumor control and favorable outcomes. The observed reduction in tumor size and the ultrasound findings suggesting complete response indicate efficacy in simultaneously targeting angiogenesis, immune evasion, and mTOR signaling. Preserved renal function and contralateral kidney hypertrophy indicate the regimen’s feasibility in selected patients. The mild toxicity profile supports the regimen’s safe administration with proper oversight. This approach offers a practical alternative in a setting where newer standard agents are unavailable.

Our findings are consistent with recent developments in the treatment of advanced RCC, particularly those using combinations of VEGF inhibitors and immune checkpoint inhibitors. For instance, the KEYNOTE-426 study demonstrated considerable survival benefits and tumor response rates in patients with advanced RCC receiving pembrolizumab in combination with axitinib (4). Similarly, phase-II trials of tislelizumab plus axitinib and toripalimab plus axitinib in the neoadjuvant setting have reported impressive objective response rates of 45% and 55.5%, respectively (5,6). VEGF inhibition can modulate the tumor microenvironment by normalizing vasculature, reducing myeloid-derived suppressor cells and regulatory T-cells, and potentially enhancing T-cell infiltration and function, thereby synergizing with PD-1 blockade (7). We added pembrolizumab to the regimen with an aim to leverage this established synergistic principle.

However, our approach differs from these therapies by incorporating additional agents – bevacizumab, sirolimus, and zoledronic acid, which target specific mechanisms of tumor progression. While bevacizumab in combination with atezolizumab has been shown to be effective in metastatic RCC (8), its role in locally advanced disease remains poorly understood. Similarly, sirolimus, an mTOR inhibitor, has demonstrated activity in ccRCC due to the frequent dysregulation of the PI3K/AKT/mTOR pathway (9). Zoledronic acid, typically used to prevent skeletal-related events in metastatic cancer (10), was used preemptively in this case, and may have contributed to the absence of skeletal complications.

We are aware that combining these agents, particularly sunitinib and bevacizumab, carries a considerable theoretical risk of cumulative and overlapping toxicities (eg, anorexia, anemia, hand-foot syndrome, mucositis, fatigue). The regimen was administered under close clinical and laboratory monitoring. The observed manageable toxicity profile in this patient suggested feasibility but cannot be extrapolated without further study. Formal pharmacokinetic and pharmacodynamic interaction studies for this five-drug combination are lacking. This approach represents an exploratory strategy based on targeting known critical pathways in ccRCC, undertaken with careful risk-benefit assessment for this high-risk patient in a context where standard combination options might have been limited. Further preclinical modeling and carefully designed early-phase clinical trials would be necessary to formally evaluate the safety and efficacy of such an intensive combination.

This case highlights the potential synergy of combining the inhibition of angiogenesis, immune modulation, and mTOR suppression, contributing to the evolving landscape of RCC combination therapies and suggesting avenues for multi-pathway research. The regimen demonstrates the potential to leverage accessible drugs for significant tumor control in resource-limited settings, with a manageable toxicity profile suitable for further evaluation and clinical adoption in appropriate contexts.

The current study has several limitations. Single-patient design limits the applicability of the approach to a broader population, and a lack of a control group prevents us from attributing the outcomes to the regimen. Combining multiple agents may pose unrecognized risks or synergistic toxicities not observed in this single case. Due to a short follow-up, long-term efficacy and survival data are lacking.While we relied solely on anatomical imaging (CT/ultrasound) for definitive complete response assessment in RCC post-treatment, these modalities, unlike functional imaging or biopsy, may not fully distinguish between treatment effect, fibrosis, and microscopic residual disease.

To build on the knowledge gained from this case, randomized studies should be conducted to assess the efficacy and safety of the regimen in larger cohorts. Furthermore, biomarker studies would be beneficial to identify predictors of response to optimize therapy. Long-term follow-up is recommended to evaluate durability and survival outcomes, while cost-effectiveness analyses should be conducted to assess economic viability in resource-limited settings.

In conclusion, this case demonstrates the efficacy of a novel multimodal regimen for locally advanced ccRCC, which achieved tumor regression and stable renal function in a resource-constrained context. By targeting multiple pathways, it offers a promising alternative to standard treatments, underscoring the value of innovation in oncology.
